# Acknowledgement of manuscript reviewers 2017

**DOI:** 10.18332/tid/87132

**Published:** 2018-03-22

**Authors:** James Elliott Scott

**Affiliations:** 1 University of Manitoba, Manitoba, Canada; 2 Pediatrics & Child Health Administrative Office, Children's Hospital, University of Manitoba, Winnipeg, Manitoba, Canada

## CONTRIBUTING REVIEWERS

The editors of Tobacco Induced Diseases would like to thank all our reviewers who have contributed to the journal in Volume 15 (2017).

**Abiola A**

Nigeria

**Abdullah ASM**

United Kingdom

**Abed Y**

Palestine

**Abidin EZ**

United Kingdom

**Abou Arbid S**

Lebanon

**Abreu-Villaça YY**

Brazil

**Abroms LC**

United States

**Abshire DA**

United States

**Abughosh SM**

United States

**Achia TN**

South Africa

**Adebiyi AO**

Nigeria

**Adsit R**

United States

**Afridi HI**

Pakistan

**Agaku I**

United States

**Agarwal A**

United States

**Aghamohammadi N**

Malaysia

**Aghazadeh S**

Malaysia

**Agnihotri R**

United States

**Agrawal A**

United States

**Aina BA**

Nigeria

**Akl EA**

Canada

**Akl EA**

United States

**Al Mulla A**

Qatar

**al’Absi M**

United States

**Alanazi OA**

United States

**Albrecht JS**

United States

**Al-Delaimy WK**

United States

**Alemán A**

Argentina

**Alemu YM**

Ethiopia

**AlHarbi BB**

United States

**Al-Houqani M**

United Arab Emirates

**Alias H**

Malaysia

**Al-Jaber A**

Saudi Arabia

**Allais L**

Belgium

**Allem JP**

United States

**Allen ML**

United States

**Almadana Pacheco V**

Spain

**Al-Matubsi HY**

Jordan

**Alpert H**

United States

**Al-Qahtani MH**

Saudi Arabia

**Al-Sadat N**

Malaysia

**Alvarado GF**

Bolivia

**Alzyoud S**

Jordan

**Amin TT**

Egypt

**Amin V**

United States

**Ammar N**

Lebanon

**Amos A**

United Kingdom

**Anand V**

United States

**Anczak JD**

United States

**Andersen MR**

Denmark

**Anderson AE**

Australia

**Andersson A**

Sweden

**Andersson C**

Sweden

**Andreas S**

Germany

**Andruszkiewicz A**

Poland

**Arab M**

Iran

**Arcury T**

United States

**Arfken CL**

United States

**Armitage CJ**

United Kingdom

**Arshad H**

United Kingdom

**Aryal UR**

Sweden

**Asare-Anane H**

Ghana

**Asfar T**

Syria

**Asfar T**

United States

**Au T**

Australia

**Auf RA**

Egypt

**Aveyard P**

United Kingdom

**Avila-Tang E**

United States

**Awan K**

United States

**Ayers JW**

United States

**Ayo-Yusuf O**

South Africa

**Azagba S**

Canada

**Babusyte A**

Lithuania

**Baca CT**

United States

**Baer JS**

United States

**Baillie E**

Australia

**Baker CJ**

United States

**Baker Holmes C**

United States

**Baltar VT**

Brazil

**Balte P**

United States

**Banerjee SC**

United States

**Bansal-Travers M**

United States

**Banzer R**

Austria

**Barnett TE**

Canada

**Barreiro E**

Spain

**Barrett E**

Australia

**Barrington-Trimis JL**

United States

**Bartlett SE**

Australia

**Bau CH**

Brazil

**Beard E**

United Kingdom

**Bektas I**

Turkey

**Belleudi V**

Italy

**Benowitz N**

United States

**Benowitz NL**

United States

**Bérard A**

Canada

**Berg CJ**

United States

**Bergen H**

Canada

**Berhane K**

United States

**Bernhard D**

Austria

**Bernstein M**

Canada

**Berrueta M**

Argentina

**Bian H**

China

**Bich NN**

Viet Nam

**Bierut LJ**

United States

**Bigna JJR**

France

**Bird Y**

Canada

**Bissonnette E**

Canada

**Blaha MJ**

United States

**Blalock JE**

United States

**Blot WJ**

United States

**Boehm G**

Austria

**Bogdanovica I**

United Kingdom

**Bolam B**

Australia

**Bondy SJ**

Canada

**Borrelli B**

United States

**Bot M**

Netherlands

**Bottorff J**

Canada

**Boudreaux ED**

United States

**Bowman J**

Australia

**Braithwaite RS**

United States

**Breysse PN**

United States

**Brimkulov N**

Kazakhstan

**Broekhuizen R**

Netherlands

**Br**ø**gger J**

Norway

**Brose LS**

United Kingdom

**Broszkiewicz M**

Poland

**Brothwell D**

Canada

**Budhwani H**

United States

**Burgess DJ**

United States

**Burkhalter JE**

United States

**Burkly LC**

United States

**Burns ME**

United States

**Byzek SA**

United States

**Cabrera L**

United States

**Cai L**

China

**Callard C**

Canada

**Canals Sans J**

Spain

**Cantor SB**

United States

**Cao S**

China

**Carlens C**

Sweden

**Carlini BH**

United States

**Carson KV**

Australia

**Carvalho CRF**

Brazil

**Castañeda-Carbajal B**

Bolivia

**Chaaya M**

Lebanon

**Chaiton MO**

Canada

**Chakrabarti G**

India

**Chamberlain CC**

Australia

**Chami HA**

United States

**Chandora R**

United States

**Chappell A**

Canada

**Chappell G**

Canada

**Chen AT**

United States

**Chen S**

China

**Chen WQ**

China

**Chen X**

United States

**Chen YH**

Taiwan

**Cheng DM**

United States

**Cheng HG**

United States

**Cheng KW**

United States

**Cheung YTD**

Hong Kong

**Chiavegato LD**

Brazil

**Chidiac JE**

United States

**Chiolero A**

Switzerland

**Chiu M**

Canada

**Cho HJ**

Republic of Korea

**Choi E**

Republic of Korea

**Choi K**

United States

**Chowdhury P**

United States

**Christophi CA**

Cyprus

**Chun J**

Republic of Korea

**Ciapponi A**

Argentina

**Cinar O**

Turkey

**Cioe PA**

United States

**Claire AW**

United States

**Clark ML**

United States

**Coelho FM**

Brazil

**Coen K**

Canada

**Cogollo-Milanés Z**

Colombia

**Cohen A**

France

**Cohen JE**

United States

**Collins BN**

United States

**Collins D**

Canada

**Collins PF**

Australia

**Colomar M**

Argentina

**Connolly G**

United States

**Coppo A**

Italy

**Corsi DJ**

Canada

**Cougle JR**

United States

**Cranford JA**

United States

**Crome E**

Australia

**Cummings KM**

United States

**Curry SJ**

United States

**Curtin GM**

United States

**Czoli C**

Canada

**Daher R**

Lebanon

**Dai H**

United States

**Dai L**

China

**Danawala SA**

United States

**Dancoine PF**

France

**Davis JM**

United States

**Dawood OT**

Malaysia

**Ddumba E**

Uganda

**De Flora S**

Italy

**de Siqueira Galil AG**

Brazil

**Dean J**

Australia

**Debigaré R**

Canada

**Dechanet C**

France

**Dede C**

Turkey

**Deen JF**

United States

**Dekker FJ**

Netherlands

**Delporte C**

Belgium

**Deressa W**

Ethiopia

**Desalu O**

Nigeria

**Dey N**

India

**Di YM**

Australia

**Dick DM**

United States

**Dickson KS**

Ghana

**Dietz NA**

United States

**Dogar O**

United Kingdom

**Dohm E**

United States

**Dolzhenko MN**

Ukraine

**Donaldson EA**

United States

**Dong Y**

China

**Doogan NJ**

United States

**Doran CM**

Australia

**Doyle GA**

United States

**Dray J**

Australia

**Drehmer JE**

United States

**Driezen P**

Canada

**Drobes DJ**

United States

**Dunlop S**

Australia

**Duperly J**

Colombia

**Durkin S**

Australia

**Dusabe-Richards JN**

United Kingdom

**Du**š**kov**á **M**

Czech Republic

**Dwivedi S**

India

**Ebbert J**

United States

**Efroymson D**

Bangladesh

**Eichner JE**

United States

**Eisenberg MJ**

United States

**El Ansari W**

United Kingdom

**El Hajj DG**

United States

**El Khoury F**

France

**Elkalmi RM**

Malaysia

**El-Sayed AM**

United States

**El-Shahawy O**

United States

**Elton-Marshall T**

Canada

**Eren G**

Turkey

**Erguder T**

Turkey

**Esteves SC**

Brazil

**Eticha T**

United States

**Everatt R**

Lithuania

**Eversman MH**

United States

**Everson-Hock ES**

United Kingdom

**Fagan P**

United States

**Falcone M**

United States

**Farber HJ**

United States

**Farshadpour F**

Netherlands

**Fatch R**

United States

**Fernander A**

United States

**Ferrante G**

Italy

**Ferrari R**

Brazil

**Figueira-Goncalves JM**

Spain

**Filion KB**

Canada

**Filippidis F**

United Kingdom

**Finegan B**

Canada

**Fitzgerald TN**

United States

**Fond G**

France

**Fong GT**

Canada

**Forbes M**

Australia

**Ford A**

United Kingdom

**Ford CA**

United States

**Foreman G**

Canada

**Forster JL**

United States

**Fotedar S**

India

**Foulds J**

United States

**Franco M**

United States

**Freeman J**

Canada

**French DP**

United Kingdom

**Fretts AM**

United States

**Freund M**

Australia

**Froelicher E**

United States

**Fu M**

Spain

**Fu SS**

United States

**Fuller W**

United States

**Furlan AJ**

United States

**Fustinoni S**

Italy

**Gaete J**

United Kingdom

**Gallopel-Morvan K**

France

**Gamarel KE**

United States

**Gao Y**

China

**Garcia-Portilla MP**

Spain

**Gentina E**

France

**Georgiou A**

Cyprus

**Gesesew H**

Australia

**Ghandour B**

Lebanon

**Ghavami S**

Canada

**Giampaoli S**

Italy

**Gilles ME**

United States

**Gilmore AB**

United States

**Girma E**

Ethiopia

**Godin G**

Canada

**Goldblatt E**

Canada

**Golden SH**

United States

**Goldstein AO**

United States

**Gong W**

China

**Gorini G**

Italy

**Gorman BK**

United States

**Gosker HR**

Netherlands

**Gottfredson NC**

United States

**Gould GS**

Australia

**Grant JD**

United States

**Green BG**

United States

**Griffis H**

United States

**Gudmundsson KG**

Iceland

**Guillaumier A**

Australia

**Guindon GE**

Canada

**Gupta A**

India

**Gupta V**

India

**Gwon SH**

United States

**Halpern-Felsher B**

United States

**Hamilton G**

New Zealand

**Hammadeh ME**

Germany

**Hammal F**

Canada

**Hammond D**

Canada

**Hamrah MH**

Afghanistan

**Han MA**

Republic of Korea

**Hanioka T**

Japan

**Hanke W**

Poland

**Hao J**

China

**Harakeh Z**

Netherlands

**Haren MT**

Australia

**Harrell MB**

United States

**Hassan LM**

United Kingdom

**Hatsukami DK**

United States

**Haubruck P**

Germany

**Hauer KE**

United States

**Hayes C**

Ireland

**He B**

China

**He N**

China

**Hefler M**

Australia

**Helgason AR**

Sweden

**Helms VE**

United States

**Helzer JE**

United States

**Henkler F**

Germany

**Hershberger AR**

United States

**Herzog TA**

United States

**Hessol NA**

United States

**Hildemann LM**

United States

**Hindocha C**

United Kingdom

**Hitchman SC**

Canada

**Hitsman B**

United States

**Ho SY**

Afghanistan

**Hock LK**

United States

**Hodder RK**

Australia

**Hoehn JL**

United States

**Hoek J**

Australia

**Holt LJ**

United States

**Holtrop JS**

United States

**Holubcikova J**

Slovakia

**Hong H**

United States

**Hood NE**

United States

**Hossain SZ**

Australia

**Howard BV**

United States

**Hoyt AT**

United States

**Hsu DJ**

United States

**Hu S**

United States

**Huang Y**

United Kingdom

**Hughes JR**

United States

**Hughes N**

Australia

**Humair JP**

Switzerland

**Hurt RT**

United States

**Hyman I**

Canada

**Impey D**

Canada

**Içmeli ÖS**

Turkey

**Irvin VL**

United States

**Itoh N**

Japan

**Jama A**

United States

**James SA**

United States

**Janzon E**

Sweden

**Jaquet A**

France

**Jarvie JA**

United States

**Jawad M**

United Kingdom

**Jensen PD**

Denmark

**Jernigan VB**

United States

**Ji Q**

China

**Jiang N**

Hong Kong

**Jiménez-Ruiz CA**

Spain

**Jin YH**

China

**John LJ**

United Arab Emirates

**Jones LL**

United Kingdom

**Jones MR**

United States

**Joung MJ**

Republic of Korea

**Jradi H**

Lebanon

**Jukema JB**

Netherlands

**Kaai S**

Canada

**Kabir MA**

Malaysia

**Kaddumukasa M**

Uganda

**Kaddumukasa MN**

Uganda

**Kaduri P**

Tanzania

**Kakde S**

United Kingdom

**Kalichman SC**

United States

**Kallischnigg G**

Switzerland

**Kamal SM**

Bangladesh

**Kang D**

Republic of Korea

**Kastaun S**

Germany

**Katsaounou P**

Greece

**Katz DA**

United States

**Kavroudakis D**

Greece

**Kayima J**

Uganda

**Keane KN**

Australia

**Kearney M**

Ireland

**Kegler M**

United States

**Kegler MC**

United States

**Kehl L**

United States

**Keith RJ**

United States

**Kelishadi R**

Iran

**Kelleher C**

Ireland

**Kengne AP**

Netherlands

**Kennedy RD**

Canada

**Khan MM**

Germany

**Khan Z**

Germany

**Khoudigian S**

Canada

**Kidane A**

Tanzania

**Kien VD**

Viet Nam

**Kilic D**

France

**Kim BG**

Republic of Korea

**Kim D**

Republic of Korea

**Kim KH**

Republic of Korea

**Kim S**

Republic of Korea

**Kim Y**

United States

**Kimbrel N**

Australia

**King B**

United States

**Kinnunen T**

United States

**Kirenga BJ**

Uganda

**Kirk GD**

United States

**Kispert S**

United States

**Kitada M**

Japan

**Kjeldgaard HK**

Norway

**Klepeis NE**

United States

**Klesges RC**

United States

**Klosky JL**

United States

**Kobayashi D**

Japan

**Kolar SK**

United States

**Korytina GF**

Russia

**Kotani K**

Japan

**Kotz D**

Netherlands

**Kotz D**

United Kingdom

**Kousoulis A**

United Kingdom

**Kowitt SD**

United States

**Kralikova E**

Sweden

**Krasovsky K**

Ukraine

**Krebs NM**

United States

**Krenek M**

United States

**Krishnan AR**

United States

**Krishnan-Sarin S**

United States

**Krukowski RA**

United States

**Kruse GR**

United States

**Kumar A**

United States

**Kuntz B**

Germany

**Kurian AK**

United States

**Kushnir V**

Canada

**Kvalvik LG**

Norway

**Lal A**

Australia

**Lam H**

Hong Kong

**Lamas CC**

Brazil

**Lancaster T**

United Kingdom

**Langen RC**

Netherlands

**Larcombe AN**

Australia

**Large M**

Australia

**Lasebikan VO**

Nigeria

**Lazicić-Putnik L**

Croatia

**Le AD**

Canada

**Lea V**

United States

**Leatherdale ST**

Canada

**Leavens E**

United States

**Lebedeva ES**

Russia

**Lee DJ**

United States

**Lee JA**

Republic of Korea

**Lee KJ**

Republic of Korea

**Lee Y**

United States

**Lee YY**

Malaysia

**Leggat P**

Australia

**Leidner AJ**

United States

**Leinsalu M**

Estonia

**Leonardi-Bee J**

United Kingdom

**Leong SL**

United States

**Lepore SJ**

United States

**Leung R**

United States

**Lewinson T**

United States

**Li L**

China

**Li S**

United States

**Li Y**

China

**Li Z**

United States

**Liang G**

China

**Liang LA**

Germany

**Liang X**

China

**Liao J**

China

**Lifson AR**

United States

**Linden K**

Finland

**Ling PM**

United States

**Lintsen AM**

Netherlands

**Lip GY**

United Kingdom

**Lippert AM**

United States

**Liu SI**

Taiwan

**Llambi L**

Argentina

**Loffredo CA**

United States

**López MJ**

Spain

**López Zubizarreta M**

Spain

**Lorenzo-Blanco EI**

United States

**Loukas A**

United States

**Lu L**

United Kingdom

**Lubin JH**

United States

**Lubman DI**

Australia

**Luger TM**

United States

**Lugoboni F**

Italy

**Luk R**

Canada

**Lund KE**

Norway

**Lynch A**

New Zealand

**Lyons M**

United States

**Macharia M**

South Africa

**Madeddu G**

Italy

**Magnusson LL**

Sweden

**Magor-Blatch LE**

Australia

**Majdzadeh R**

Iran

**Makin JK**

Australia

**Malaguti C**

Brazil

**Malcon MC**

Brazil

**Mamudu HM**

United States

**Mandil A**

Egypt

**Mandil A**

Saudi Arabia

**Manivong P**

Canada

**Manning KC**

United States

**Mantey DS**

United States

**Manzoli L**

Italy

**Marano KM**

United States

**Marcano-Belisario JS**

United Kingdom

**Mariano G**

United States

**Marsh EE**

United States

**Marsh L**

New Zealand

**Martinez E**

United States

**Martinez P**

Norway

**Martinez-Donate A**

United States

**Martinez-Novack MC**

Bolivia

**Martínez-Sánchez JM**

Spain

**Marynak KL**

United States

**Massey SH**

United States

**Matsumoto A**

United States

**Matsuo K**

Japan

**Matt GE**

United States

**Maynard OM**

United Kingdom

**Maziak W**

United States

**Mbah AK**

United States

**Mbulo L**

United States

**McBride CM**

United States

**McCabe SE**

United States

**McCaffery JM**

United States

**McCarty MC**

United States

**McConnell R**

United States

**McDaniel P**

United States

**McEvoy CT**

United States

**McKenzie M**

Australia

**McLellan DL**

United States

**Meenakshisundaram R**

India

**Meenawat A**

India

**Meghea CI**

United States

**Mehari A**

United States

**Meier EM**

United States

**Melani AS**

Italy

**Meltzer LR**

United States

**Menezes AM**

Brazil

**Mensah GA**

United States

**Mia MN**

Bangladesh

**Michal M**

Germany

**Michel L**

France

**Milanzi EB**

Netherlands

**Milcarz K**

Poland

**Millan DS**

United Kingdom

**Mills B**

Australia

**Mills KL**

Australia

**Minaker LM**

Canada

**Minh HV**

Viet Nam

**Miranda JJ**

Peru

**Mishra GA**

India

**Mitchell F**

United Kingdom

**Mohamed NN**

Malaysia

**Mohammad Poorasl A**

Iran

**Mohan A**

India

**Moher D**

Canada

**Mokdad AH**

United States

**Molina AJ**

Spain

**Montreuil A**

Canada

**Moodie C**

United Kingdom

**Moore GF**

United Kingdom

**Morello P**

Argentina

**Morjaria JB**

United Kingdom

**Moshammer H**

Austria

**Moyses C**

Australia

**Mpabulungi L**

Uganda

**Mufunda J**

Eritrea

**Mugenyi L**

Uganda

**Muranaka N**

United States

**Murdoch DM**

United States

**Murphy-Hoefer R**

United Kingdom

**Mushtaq N**

United States

**Mutchinick OM**

Mexico

**Muula AS**

United States

**Naddafi K**

Iran

**Nagaya T**

Japan

**Nagelhout GE**

Netherlands

**Nahar VK**

United States

**Nahvi S**

United States

**Nakhaee N**

Iran

**Nakkash R**

Lebanon

**Nam GE**

Republic of Korea

**Narbutaite J**

Lithuania

**Nargis N**

United States

**Nazar GP**

United Kingdom

**Neas BR**

United States

**Neblett RC**

United States

**Nemakhavhani TR**

South Africa

**Nepal S**

Australia

**Newhall K**

Lebanon

**Nguyen HV**

Canada

**Niebauer J**

Austria

**Niemiec CP**

United States

**Nishiyama M**

Japan

**Nong G**

China

**Noor Hassim I**

Malaysia

**Nturibi EM**

Kenya

**Nyberg A**

Canada

**O’Connor RJ**

United States

**Obeidat SR**

Jordan

**O’Carroll H**

Ireland

**Ockene JK**

United States

**Odgerel Z**

United States

**Oger P**

France

**Oh K**

Republic of Korea

**Oh YM**

Republic of Korea

**O’Halloran KD**

Ireland

**Ojo-Fati O**

United States

**Okello S**

Uganda

**O’Leary R**

Canada

**Oliveira Serra MAA**

Brazil

**Omoloja A**

United States

**Odukoya OO**

Nigeria

**Ortiz-Ortiz MT**

Bolivia

**Ossip D**

United Kingdom

**Osazuwa-Peters N**

United States

**Osman A**

United States

**Owusu-Dabo E**

United Kingdom

**Pacek L**

United States

**Palipudi KM**

United States

**Papadakis S**

Canada

**Parchwani DN**

India

**Park HK**

United States

**Park J**

Republic of Korea

**Park S**

Republic of Korea

**Park WJ**

Republic of Korea

**Patel D**

United States

**Patelarou E**

United Kingdom

**Pavia M**

Italy

**Payne JD**

United States

**Peck KR**

United States

**Peckham E**

United Kingdom

**Pedersen W**

Norway

**Pederson A**

Canada

**Pellowski JA**

United States

**Peltzer K**

South Africa

**Pennanen M**

Finland

**Pérez-Milena A**

Spain

**Perkins KA**

United States

**Perkins R**

United Kingdom

**Perret JL**

Australia

**Perry CL**

United States

**Petersen AB**

United States

**Philip NS**

United States

**Pines H**

United States

**Pinget C**

Switzerland

**Piper ME**

United States

**Pittilo MR**

United Kingdom

**Pobutsky A**

United States

**Pohoreski K**

Canada

**Pokhrel P**

United States

**Polanska K**

Poland

**Polkey M**

United Kingdom

**Pollack HA**

United States

**Poluyi EO**

Nigeria

**Popova L**

United States

**Poulet E**

France

**Powers CA**

United States

**Prabhakaran D**

India

**Pradhan PM**

Nepal

**Pretorius E**

Australia

**Price P**

Canada

**Primack BA**

United States

**Prochaska J**

United States

**Qian ZB**

China

**Quadri MF**

Saudi Arabia

**Quinn VP**

United States

**Rahmanian SD**

United States

**Rajkumar S**

United States

**Ramanandraibe N**

Congo

**Ramchandani VA**

Australia

**Ramo DE**

United States

**Rasmussen L**

United States

**Raspanti GA**

United States

**Ravara SB**

United States

**Razaz-Rahmati N**

Canada

**Record R**

United States

**Reda AA**

United States

**Reid RD**

Canada

**Reinhold B**

United States

**Reisen CA**

United States

**Rezaei S**

Iran

**Richardson JL**

United Kingdom

**Righetti RF**

Brazil

**Rizov M**

United Kingdom

**Roberts B**

United Kingdom

**Roberts G**

United Kingdom

**Rock VJ**

United States

**Rodu B**

United States

**Rogalska-Płońska M**

Poland

**Roman NV**

South Africa

**Romer D**

United States

**Romero Paredes MD**

Spain

**Roos A**

Canada

**Rose DE**

United States

**Rose GL**

United States

**Rosen LJ**

Israel

**Rossheim ME**

United States

**Rowe SM**

United States

**Royo-Bordonada M**Á

Spain

**Rudatsikira E**

United States

**Ruhil R**

India

**Ruprecht AA**

Italy

**Ryu JS**

Republic of Korea

**Ryu SY**

Republic of Korea

**Saade G**

Lebanon

**Sagar M**

United States

**Sairu P**

India

**Saito A**

Japan

**Sajatovic M**

United States

**Samet J**

United States

**Samet JH**

United States

**Santus P**

Italy

**Savard J**

Canada

**Schick SF**

United States

**Schlagintweit HE**

Canada

**Schmelzle J**

Canada

**Schols AM**

Netherlands

**Schootman M**

United States

**Schwartz JL**

United States

**Scott D**

United States

**Sebrié EM**

United States

**Segan C**

Australia

**Selby P**

Canada

**Semakula HM**

Belgium

**Seo DC**

United States

**Sargent JD**

Mexico

**Serror K**

France

**Seymour JM**

United Kingdom

**Shah BR**

Canada

**Shah C**

India

**Shalaby NM**

Egypt

**Shelley D**

United States

**Shenassa ED**

United States

**Sherman S**

United States

**Sherriff NS**

United Kingdom

**Shetty K**

United States

**Shi Z**

China

**Shim IH**

Republic of Korea

**Shivanna B**

United States

**Shtarkshall RA**

Israel

**Sidani JE**

United States

**Simpson CA**

United States

**Singh RF**

United States

**Singh SK**

Malaysia

**Singh T**

United States

**Sirirassamee T**

Thailand

**Smith D**

Australia

**Smith JJ**

United States

**Smith KM**

United States

**Smith RC**

United States

**Smyrnakis E**

Greece

**Soboka M**

Ethiopia

**Sofuoglu M**

United States

**Solé D**

Brazil

**Song SM**

Republic of Korea

**Song Y**

United States

**Soulakova JN**

United States

**Soule EK**

United States

**Spindle TR**

United States

**Sreeramareddy CT**

United States

**Stead M**

United Kingdom

**Steer J**

United Kingdom

**Stein JH**

United States

**Stepankova L**

Czech Republic

**Stepanov I**

United States

**Sterling K**

United States

**Stevens M**

Australia

**Stillman FA**

United States

**Stock C**

Denmark

**Stotts AL**

United States

**Streel S**

Belgium

**Ström JO**

Sweden

**Subramaniam M**

Singapore

**Subramanian S**

India

**Sulsky SI**

United States

**Sussman S**

United States

**Sutfin EL**

United States

**Svedsater H**

United Kingdom

**Swallow EB**

United Kingdom

**Sweanor D**

Canada

**Taber J**

United States

**Tachfouti N**

Morocco

**Tait EM**

United States

**Talati A**

United States

**Tan AS**

United States

**Tan S**

United States

**Tang AM**

United States

**Tang K**

United States

**Tang Y**

China

**Tao ZZ**

China

**Tautolo el-S**

New Zealand

**Taype-Rondan A**

Peru

**Tchicaya A**

Luxembourg

**Teesson M**

Australia

**Tehranifar P**

United States

**Teklehaimanot HD**

Ethiopia

**Temple B**

Canada

**Tesoriero JM**

United States

**Theodoro-Júnior OA**

Brazil

**Thomas D**

Australia

**Thompson ME**

Canada

**Thomson G**

New Zealand

**Tikkanen M**

Finland

**To Y**

Japan

**Tolstrup JS**

Denmark

**Tomar SL**

United States

**Tønnesen P**

Denmark

**Torchalla I**

Canada

**Towns S**

Australia

**Trigwell J**

United Kingdom

**Troosters T**

Belgium

**Trzeciak BG**

Poland

**Tucker JA**

United States

**Tulloch HE**

Canada

**Tumwine J**

Uganda

**Umbricht A**

United States

**Underner M**

France

**Unger J**

United States

**Urman R**

United States

**Ussher M**

United Kingdom

**Valença SS**

Brazil

**van Gemert F**

Netherlands

**Van Minh H**

Viet Nam

**Vander Weg M**

United States

**Vanderkam P**

France

**Vanuzzo D**

Italy

**Varkey B**

United States

**Vayá A**

Spain

**Vellios N**

South Africa

**Vercruysse GA**

United States

**Vermeeren MA**

Netherlands

**Vidal-Cardenas E**

United States

**Viriyachaiyo V**

Thailand

**Visseren FLJ**

Netherlands

**Vivilaki V**

Greece

**von Garnier C**

Switzerland

**Vonk JM**

Netherlands

**Wagener TL**

United States

**Wagijo MA**

Netherlands

**Wakefield M**

Australia

**Walker N**

New Zealand

**Walters J**

Australia

**Wang K**

United States

**Wang L**

China

**Wang XL**

United States

**Wang Y**

United States

**Wang Z**

China

**Ward KD**

United States

**Warren CW**

United States

**Wassum K**

United States

**Wdowiak A**

Poland

**Webb MS**

United States

**Webb Hooper M**

United States

**Wee LH**

Malaysia

**Weiland T**

Australia

**Weiss JW**

United States

**Weissman MM**

United States

**Welsh DA**

United States

**West O**

United Kingdom

**Westergaard CG**

Denmark

**Wetherill MS**

United States

**Wetter DW**

United States

**Weygandt PL**

United States

**White C**

Canada

**Wiener RC**

United States

**Wiggers J**

Australia

**William Li HC**

China

**Willis D**

United States

**Winickoff JP**

United States

**Winkles JA**

United States

**Wolfenden L**

Australia

**Wong LP**

Malaysia

**Woolhouse H**

Australia

**Wu M**

China

**Wang XL**

United States

**Xu M**

China

**Xu Y**

China

**Yan J**

China

**Yan XS**

United States

**Yang H**

China

**Yang L**

China

**Yang T**

China

**Yang Y**

China

**Yao He**

China

**Yassin N**

Lebanon

**Ye X**

China

**Yeh F**

United States

**Yeung EH**

United States

**Yildirim AÖ**

Germany

**Yim A**

United States

**Yingst JM**

United States

**Yong HH**

Australia

**Yoong SL**

Australia

**Younes N**

Qatar

**Yu B**

United States

**Yun JH**

United States

**Yusuf K**

Canada

**Zaninotto P**

United Kingdom

**Zatonski W**

Poland

**Zaw KK**

Myanmar

**Zawertailo LA**

Canada

**Zgaga L**

Ireland

**Zhang HL**

China

**Zhang J**

China

**Zhang X**

United States

**Zhang Y**

China

**Zhang Z**

China

**Zhao L**

United States

**Zheng LY**

United States

**Zheng P**

China

**Zhong J**

China

**Zhou S**

China

**Zhu Z**

China

**Zirakzadeh A**

United States

**Zorlu N**

Turkey

**Zuo JJ**

China

**Zyoud SH**

Palestine

